# The Pol IV largest subunit CTD quantitatively affects siRNA levels guiding RNA-directed DNA methylation

**DOI:** 10.1093/nar/gkz615

**Published:** 2019-07-22

**Authors:** Jered M Wendte, Jeremy R Haag, Olga M Pontes, Jasleen Singh, Sara Metcalf, Craig S Pikaard

**Affiliations:** 1 Department of Biology, Indiana University, 915 E. Third Street, Bloomington, IN 47405, USA; 2 Division of Biology and Biomedical Sciences, Washington University, St Louis, MO 63130, USA; 3 Department of Molecular and Cellular Biochemistry, Indiana University, 915 E. Third Street, Bloomington, IN 47405, USA; 4 Howard Hughes Medical Institute, Indiana University, 915 E. Third Street, Bloomington, IN 47405, USA

## Abstract

In plants, nuclear multisubunit RNA polymerases IV and V are RNA Polymerase II-related enzymes that synthesize non-coding RNAs for RNA-directed DNA methylation (RdDM) and transcriptional gene silencing. Here, we tested the importance of the C-terminal domain (CTD) of Pol IV’s largest subunit given that the Pol II CTD mediates multiple aspects of Pol II transcription. We show that the CTD is dispensable for Pol IV catalytic activity and Pol IV termination-dependent activation of RNA-DEPENDENT RNA POLYMERASE 2, which partners with Pol IV to generate dsRNA precursors of the 24 nt siRNAs that guide RdDM. However, 24 nt siRNA levels decrease ∼80% when the CTD is deleted. RNA-dependent cytosine methylation is also reduced, but only ∼20%, suggesting that siRNA levels typically exceed the levels needed for methylation of most loci. Pol IV-dependent loci affected by loss of the CTD are primarily located in chromosome arms, similar to loci dependent CLSY1/2 or SHH1, which are proteins implicated in Pol IV recruitment. However, deletion of the CTD does not phenocopy *clsy* or *shh1* mutants, consistent with the CTD affecting post-recruitment aspects of Pol IV activity at target loci.

## INTRODUCTION

Multisubunit RNA polymerases IV and V are plant-specific enzymes that play non-redundant roles in RNA-directed DNA methylation (RdDM), a process important for silencing DNA viruses, transposons, repetitive elements and a subset of genes (reviewed in: 1,2). In partnership with RDR2, Pol IV generates precursor RNAs, averaging only ∼32 bp in length (3–6), that are cleaved by the Dicer endonuclease, DCL3 to produce 24 nt siRNAs (3,7,8). The siRNAs are then loaded into an Argonaute family protein, primarily AGO4 or AGO6 (9,10) and guide the siRNA–AGO complexes to sites of Pol V transcription (11–13). In subsequent steps that are not well understood, the AGO–siRNA–Pol V transcription complexes mediate recruitment of chromatin modifying activities that include the cytosine methyltransferase, DRM2, resulting in extensive *de novo* cytosine methylation and associated histone modifications that collectively repress transcription by Pols I, II or III (14–16).

Pols IV and V are each composed of twelve subunits, most of which are encoded by the same genes as Pol II subunits (17). Subunits of Pols IV and/or V that differ from those of Pol II are encoded by genes that arose through duplication or retrotransposition of the ancestral Pol II subunit genes (18,19). Among the latter are the genes encoding the Pol IV and Pol V largest subunits, NRPD1 and NRPE1, respectively. Multiple exon–intron junction positions within the *NRPD1* and *NRPE1* genes are identical to those in *NRPB1*, which encodes the Pol II largest subunit, indicative of their shared ancestry (19). However, the 3′ ends of the NRPB1, NRPD1 and NRPE1 proteins are distinct. *NRPB1* encodes a C-terminal domain (CTD) composed of a repeating seven amino acid motif that is highly conserved throughout eukaryotes (20). These heptad repeats are missing in Pols IV and V (19,21,22). Instead, the Pol IV largest subunit has a relatively short CTD consisting almost entirely of a Defective Chloroplasts and Leaves (DeCL) subdomain named for its sequence similarity to a small protein family implicated in chloroplast pre-rRNA processing (23,24). A similar, but non-identical DeCL subdomain is present in the Pol V largest subunit CTD as one of at least four recognizable subdomains. Three of these subdomains affect Pol V function at different subsets of target loci (19,21,22,25), but deletion of the DeCL subdomain causes Pol V transcripts to become undetectable and eliminates nearly all Pol V-dependent RdDM (25). Importantly, Pol V transcription *in vitro* is unaffected by deletion of the DeCL subdomain (25). Collectively, these results have suggested that the DeCL subdomain of Pol V may be required for the enzyme's recruitment or activity in the context of chromatin. Consistent with this interpretation, loci that are mis-regulated in the absence of the NRPE1 DeCL subdomain overlap considerably with loci that are mis-regulated in mutants defective for proteins implicated in Pol V recruitment to target loci (25).

Based on the prior studies of Pol V, a reasonable hypothesis is that the DeCL domain of Pol IV might be important for Pol IV recruitment to target loci, which is thought to be mediated by members of the CLASSY (CLSY) protein family (CLSY1 through CLSY4) and SAWADEE HOMEODOMAIN HOMOLOG 1 (SHH1; also known as DTF1) (5,26–28). CLSY proteins are predicted to be adenosine triphosphate (ATP)-dependent DNA translocases, a protein superfamily that includes chromatin remodelers, helicases and DNA repair enzymes (29). CLSY1 and CLSY2 primarily enable Pol IV function at loci interspersed among genes in the chromosome arms whereas CLSY3 and CLSY4 mostly facilitate Pol IV function within pericentromeric regions that are gene-poor and composed of dense heterochromatin (27). SHH1/DTF1 has a domain that binds Histone H3 dimethylated on lysine 9 (H3K9me2) and is thought to recruit Pol IV to target sites enriched for this modification (26,27) in crosstalk with CG maintenance methylation, dependent on DNA methyltransferase MET1 and histone deacetylase HDA6 (30). The activities of MET1 and HDA6 allow for transgenerational epigenetic inheritance of Pol IV recruitment signals (30). Loci requiring SHH/DTF1 are enriched within chromosome arms, similar to CLSY1-2-dependent loci, and SHH1/DTF1 and CLSY1 interact in yeast two-hybrid assays, suggesting that they work in partnership (28).

In this study, we tested the importance of the Pol IV CTD for Pol IV transcription and termination in vitro, as well as the functional coupling of Pol IV and RDR2 activities, which act sequentially to generate the dsRNA precursors of 24 nt siRNAs. We also tested the importance of the CTD for siRNA biogenesis, cytosine methylation and transcriptional silencing, which are the molecular phenotypes indicative of Pol IV function *in vivo*. We show that in the absence of the CTD, Pol IV transcription, RDR2 association, Pol IV termination and Pol IV-RDR2 transcriptional coupling are all unimpaired in vitro, indicating that the CTD is dispensable for the known catalytic activities of Pol IV. Nonetheless, 24 nt siRNA levels and cytosine methylation are reduced *in vivo*. Like methylated loci dependent on SHH1 and CLSY1/2, loci dependent on the Pol IV CTD are more prevalent within chromosome arms than in pericentromeric regions. However, the molecular phenotypes resulting from deletion of the Pol IV CTD differ from those of *shh1* and *clsy1clsy2*, suggesting that the CTD affects steps of the Pol IV transcription cycle other than, or in addition to, recruitment to target loci.

## MATERIALS AND METHODS

### Plant material

*Arabidopsis thaliana* mutants *nrpd1-3* and *nrpe1-11* have been described previously (22,31), as have full length NRPD1-FLAG (*nrpd1-3*) and NRPB2-FLAG (*nrpb2*) transgenes and *nrpb2* (32,33). All plants were grown in soil in long day conditions (16 h light, 8 h dark).

### Generation of the NRPD1ΔCTD transgenic line

The C-terminal domain deletion of NRPD1 (amino acids 1337–1453) was generated by modification of a pENTR-NRPD1 full-length genomic clone with the native gene promoter (33). Using this clone as the DNA template, polymerase chain reaction (PCR) amplification of the gene without the CTD (Supplementary Table S8) was accomplished using Pfu Ultra DNA polymerase (Stratagene). The PCR product was cloned into pENTR-TOPO S/D (Invitrogen) then recombined into pEarleyGate302 to generate a C-terminal FLAG epitope fusion (34). The plasmid was transformed into *Agrobacterium tumefaciens* and used to transform *nrpd1-3* plants using the floral dip method (35,36).

### Protein immunoprecipitation and immunoblot analysis

Proteins were extracted from 4 grams of ∼2.5 week-old above-ground plant tissues ground to a fine powder in liquid nitrogen. The resulting powder was suspended in 14 ml lysis buffer (50 mM Tris HCl pH 7.6, 150 mM NaCl, 5mM MgCl_2_, 10% glycerol, 0.5 mM Dithiothreitol (DTT), 0.1% IGEPAL, 1% plant protease inhibitors (Sigma)), filtered through two layers of Miracloth and subjected to centrifugation at 16 000 × *g*, 15 min, 4°C. The resulting supernatant was incubated with anti-FLAG Agarose (Sigma) for 2 h at 4°C on a rotating mixer. The agarose resin was then washed twice with lysis buffer and boiled in sodium dodecyl sulphate-polyacrylamide gel electrophoresis (SDS-PAGE) sample buffer. SDS-PAGE was conducted using Tris-glycine gels and proteins were then transferred to nitrocellulose membranes for immunoblotting. Antibodies were diluted in TBST + 5% (w/v) nonfat dried milk as follows: 1:500 anti-NRP(D/E)2, 1:250 anti-RDR2, 1:500 anti-NRP(B/D/E)11 and 1:2000 anti-FLAG-HRP (Sigma). Anti-rabbit-HRP (Santa Cruz Biotechnology) diluted 1:5000 was used as secondary antibody to bind the primary antibodies. Native antibodies to NRP(D/E)2, RDR2 and NRP(B/D/E)11 were previously described (25,31,37).

### Nuclear immunolocalization

Immunolocalization studies were conducted as described previously (Pontes *et al.*, (33)), using nuclei of 4-week-old plants fixed in 4% formaldehyde and antibodies recognizing the C-terminal FLAG epitopes fused to the recombinant NRPD1 proteins.

### *In* *vitro* transcription assays

Equimolar amounts of template DNA, non-template DNA and RNA primer, whose sequences are provided in Supplementary Figure S1, were mixed in annealing buffer (30 mM HEPES-KOH at pH 7.6, 100 mM potassium acetate), brought to a boil in a water bath and slowly cooled to room temperature. For Pol IV reactions involving end-labeled primer RNA, a 10% excess of non-template DNA was used to ensure the complete annealing of the RNA primer. End-labeling was achieved using T4 polynucleotide kinase (T4 PNK, NEB) and 25 μCi of [γ-^32^P]-ATP (6000 Ci/mmol; Perkin Elmer).

*In vitro* transcription reactions involved Pol IV–RDR2 complexes affinity captured on anti-FLAG agarose resin (Sigma) (4,25). A total of 25 μl of polymerase-bound resin was washed once with low salt buffer (100 mM potassium acetate, 25 mM HEPES-KOH pH 7.9, 20% glycerol, 0.1 mM ethylenediaminetetraacetic acid (EDTA), 0.5 mM DTT, 1 mM PMSF), then adjusted to 50 μl in low salt buffer buffer. A total of 50 μl of 2× transcription reaction buffer (template nucleic acids, 120 mM ammonium sulfate, 40 mM HEPES-KOH pH 7.6, 20 mM magnesium sulfate, 20 μM zinc sulfate, 20% glycerol, 0.16 U/μl RNaseOUT, 20 mM DTT) was added to the 50 μl of resin slurry with associated RNA polymerases or non-specifically associated proteins of non-transgenic plant lysate controls. For end-labeled primer extension assays, the final template concentration was 25 nM and for the body-labeling assay, the template concentration was 250 nM. The final nucleotide triphosphate (NTP) concentration for end-labeling assay was 1 mM each of ATP, GTP, CTP and UTP and for the body labeling assay it was 1 mM each of GTP, CTP and UTP, 40 μM ATP and 10 μCi of [α-^32^P]-ATP (3000 Ci/mmol; Perkin Elmer). Transcription reactions were conducted at room temperature for 60 min on a rotating mixer and stopped by addition of 20 mM EDTA and heating at 75°C for 5 min. Transcription products were desalted using PERFORMA spin columns (EdgeBio) and precipitated using 1/10 volume of 3M sodium acetate, pH 5.2, 20 μg glycogen and three volumes of isopropanol at −20°C overnight. Radioactive RNA transcripts were resolved on 15% denaturing polyacrylamide gels, transferred to Whatman 3MM filter paper, dried under vacuum and visualized by autoradiography.

### Whole genome bisulfite sequencing and analysis

Bisulfite sequencing and analysis was conducted as described in (25). DNA (100 ng) extracted from ∼2.5-week-old above ground plant tissues was prepared using the Illumina TruSeq DNA methylation library prep kit according to the manufacturer's instructions. Libraries were sequenced using an Illumina NextSeq instrument. Base calling, adapter trimming, and read size selection (≥35 bp) were performed using bcl2fastq v2.16.0.10. To counter methylation end bias, the first seven and last two bases of each read were removed and a minimum q-score of 25 was applied as a filter using Cutadapt version 1.9.1 (38). Mapping of sequenced reads to the *A. thaliana* TAIR10 genome, removal of PCR duplicates and extraction of methylation information for cytosines with a minimum coverage of five reads was completed using Bismark version 0.16.1 with default settings (39). The bisulfite conversion rate was calculated based on the number of methylated cytosines divided by total number of mapped cytosines (converted and un-converted) of the chloroplast genome, which is unmethylated.

Differently methylated regions (DMRs) for CHH methylation relative to Col-0 were defined using the R package methylKit version 0.9.5 (40). The genome was analyzed in 300 bp sliding windows with a step size of 200 bp. Windows were counted if 10 informative cytosines with at least five reads each were observed. Significant hypo-DMRs, where methylation is reduced relative to wild-type (WT), were assessed at a cutoff value of at least a 10% decrease relative to WT. DMRs were calculated using a logistic regression and significance was assumed for q-values ≤ 0.01, with *P*-values corrected for multiple testing using the SLIM method (40). Changes in methylation between genotypes and statistic values were calculated using both biologic replicates to account for variation between replicates. Overlapping DMRs were merged into a single region prior to determining the number of DMRs in each line. Only DMRs that also met minimum coverage criteria in our sRNA sequencing data sets (see ‘sRNA analysis’ section) were considered in the analyses in Figures 2, 3 and 6.

To quantify % methylation within regions of interest, the methylKit regionCounts function was utilized with the genomic coordinates of interest input as a bed file (40). In box plots, outliers 1.5 times the interquartile range beyond the upper or lower quartile were omitted for visualization purposes but were included in all calculations. For heatmap and hierarchical clustering analyses, the heatmap.2 R function was utilized with default clustering settings (http://www.R-project.org/). To find the distribution of DMRs between euchromatin and dense heterochromatin, as shown in Figure 6D, the following coordinates were used to designate the dense pericentromeric heterochromatin regions on each chromosome: Chr1: 12150001-17900000; Chr2: 1000001-7350000; Chr3: 10600001-16350000; Chr4: 2550001-6400000; Chr5: 9550001-15050000. Regions falling within the heterochromatic knob of Chromosome 4 (Chr4: 1300001-2400000) were also considered dense heterochromatin regions.

To calculate % methylation values for the locus-specific analyses of Figures 4 and 5, % methylation was summed for all Pol IV-dependent DMRs, regardless of small RNA coverage, found to overlap the locus of interest, including 300 bp of 5′ and 3′ flanking sequence.

Accession numbers for previously published bisulfite sequencing data are provided in Supplementary Table S1.

### sRNA analysis

Whole genome small RNA sequencing was conducted using 1 μg total RNA isolated from 2.5-week-old above-ground plant tissues isolated using Trizol. Libraries were prepared using the Illumina TruSeq small RNA library prep kit according to the manufacturer's instructions, except that the size selection step was adjusted to select for RNAs of 15–60 nt.

Raw reads were adapter and quality (q > 20) trimmed and size selected (16–60 nucleotides) using Cutadapt version 1.9.1 (38). Reads were first filtered to remove structural RNAs (tRNAs, rRNAs, snRNAs and snoRNAs), then mapped to the *Arabidopsis* TAIR10 genome using ShortStack version 3.4 default settings (41).

Small RNA read counts for regions of interest were extracted from BAM files using the ShortStack –locifile function (41). First, read counts for all Pol IV-dependent CHH DMRs identified from bisulfite sequencing data (see above) were obtained for both Col-0 replicates. Only those regions that had a minimum of 25 reads, were classified as 24 nt clusters (minimum of 80% of reads being the 24 nt size class) in both Col-0 replicates, and showed a minimum −2 log_2_ fold change in *nrpd1-3* mutants were used for the sRNA the DNA methylation analyses in Figures 2, 3 and 6. Counts were normalized as reads per million (RPM) bases of total mapped reads. To calculate the log_2_ fold change relative to Col-0, 0.5 reads were first added to the read count for each region of interest to account for 0 values. After read count normalization, the average RPM of WT Col-0 was calculated and log_2_ fold changes were calculated for each sample as: log_2_ (Sample RPM/Avg. WT Col-0 RPM). In the Venn diagram in Figure 2C, the numbers represent the average log_2_ fold change of both replicates for each line. In box plots, outliers 1.5 times the interquartile range beyond the upper or lower quartile were omitted for visualization purposes but included in all calculations. For heatmap and hierarchical clustering analyses, the heatmap.2 R function was utilized with default clustering settings (http://www.R-project.org/). To find the distribution of regions between euchromatin and dense heterochromatin, as shown in Figure 6C, the following coordinates were used to designate the dense pericentromeric heterochromatin regions on each chromosome: Chr1: 12150001-17900000; Chr2: 1000001-7350000; Chr3: 10600001-16350000; Chr4: 2550001-6400000; Chr5: 9550001-15050000. Regions falling within the heterochromatic knob of Chromosome 4 (Chr4: 1300001-2400000) were also considered dense heterochromatin regions. Accession numbers for previously published sRNA data are listed in Supplementary Table S1.

To analyze Pol IV dependent precursor RNAs, as shown in Figure 3F and Supplementary Table S7, read sizes of 26–60 nt were summed for each Pol IV DMR in each Col-0 replicate and only those regions with a minimum coverage of 10 reads for precursor RNA size classes in both Col-0 replicates were analyzed for each genotype.

To calculate log_2_ fold change values for the locus-specific analyses in Figures 4 and 5, 24 nt siRNA clusters were identified genome-wide. The 24 nt siRNA clusters were defined with a minimum coverage of 25 reads, comprised of at least 80% 24 nt siRNAs. Clusters within 75 bp were merged into a single region. Only clusters that were identified in both Col-0 WT replicates were analyzed. Read counts were summed in each replicate of each genotype for all 24 nt siRNA clusters found to overlap the locus of interest with 300 bp of 5′ and 3′ flanking sequences included and log_2_ fold changes were calculated relative to the average Col-0 RPM as described above.

### Chop PCR

Genomic DNA was double-digested with AluI and HaeIII (NEB) at 37°C for 3 h, followed by PCR using GoTaq Green (Promega) with primers that flank enzyme cut sites listed in Supplementary Table S8. For no-digest controls, DNA was subjected to the same protocol but with no restriction enzymes added.

### RT-PCR

RNA extraction of 2.5 weeks above-ground tissues was conducted using Trizol. Reverse transcription was conducted using SuperScript III (Invitrogen), 5 μg RNA and random primers (Sigma) PCR amplification was conducted using GoTaq Green (Promega) with primers listed in Supplementary Table S8.

## RESULTS

### The NRPD1 CTD is dispensable for Pol IV subunit assembly, nuclear localization, RNA polymerase activity and transcriptional coupling with RDR2

**Figure 1.**
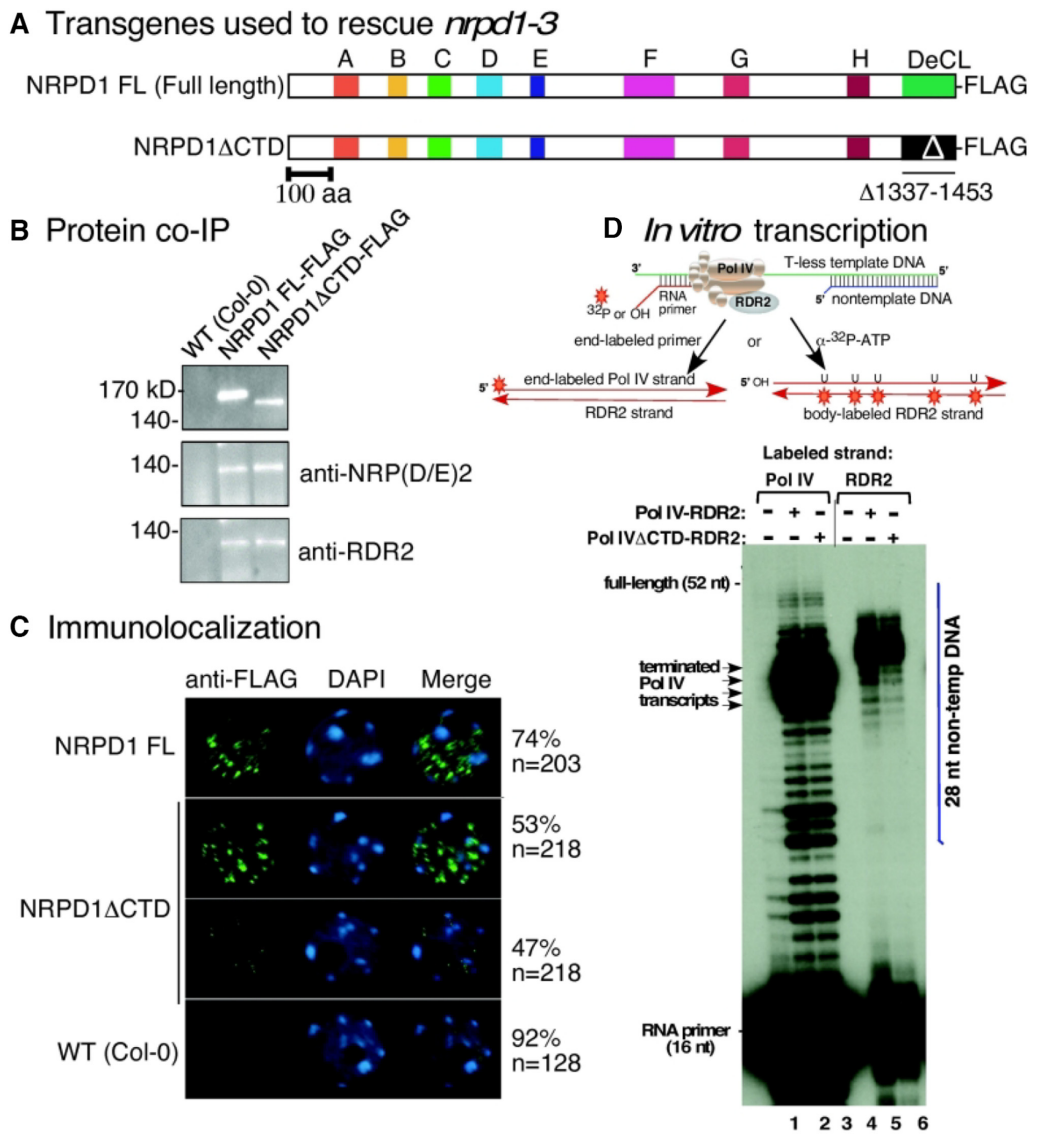
Expression and functional analysis of full length *NRPD1* and NRPD1ΔCTD transgenes. (**A**) Schematic of the full length NRPD1 protein and NRPD1 missing the C-terminal DeCL domain. Domains A-H are conserved in all multi-subunit RNA polymerase largest-subunit genes. NRPD1 has a unique carboxy-terminal domain (CTD) characterized by a Defective Chloroplasts and Leaves (DeCL) domain similar to proteins involved in chloroplast function. Full length NRPD1 and NRPD1 with the DeCL domain deleted (deletion encompassing amino acids 1337–1453, referred to as NRPD1ΔCTD) were transformed into *nrpd1-3* mutant plants. Both constructs incorporated a C-terminal FLAG epitope. (**B**) Western blot of anti-FLAG immunoprecipitated full length NRPD1 or NRPD1ΔCTD proteins. The top panel was probed using anti-FLAG antibodies. The middle and bottom panels were probed using antibodies that recognize the Pol IV second-largest subunit, NRP(D/E)2 or the Pol IV interacting protein RNA DEPENDENT RNA POLYMERASE 2 (RDR2), respectively. Non-transgenic Col-0 was also subjected to immunoprecipitation as a negative control. (**C**) Formaldehyde-fixed nuclei were incubated with anti-FLAG antibodies to detect recombinant NRPD1 proteins. Nuclei of non-transgenic Col-0 serve as a negative control. The number of nuclei examined, and the frequency of localization patterns resembling the representative images shown, are provided to the right of the images. (**D**) The NRPD1 CTD has no effect on Pol IV transcription elongation, termination or RDR2 coupling. Affinity purified Pol IV–RDR2 complexes assembled using full-length NRPD1 or NRPD1ΔCTD were tested for both Pol IV and RDR2 activity using a 52 nt T-less template DNA annealed to a 28 nt nontemplate DNA strand and 16 nt RNA primer, as depicted in the cartoon. In lanes 1–3, transcription reactions were conducted using ^32^P end-labeled RNA primer, allowing first strand transcripts generated by Pol IV to be visualized following denaturing polyacrylamide gel electrophoresis and autoradiography. In lanes 3–6, an unlabeled RNA primer was used to initiate first strand synthesis and α-^32^P-ATP incorporation was used to body- label second strands synthesized by RDR2. In lanes 1 and 3, fractions resulting from mock FLAG affinity purification of proteins from lysates of non-transgenic plants were tested as negative controls. The position of the nontemplate DNA strand, relative to the transcripts, is depicted by the blue vertical bar to the right of the autoradiogram. Nucleic acid sequences are provided in Supplementary Figure S1B.

Transgenes expressing full length NRPD1 or NRPD1 lacking the C-terminal DeCL domain (amino acid positions 1337–1453; referred to as NRPD1ΔCTD; Figure 1A) were transformed into *nrpd1-3*, a null mutant with a T-DNA insertion disrupting the Pol IV largest subunit gene. FLAG epitope tags fused to the C-termini of the recombinant NRPD1 proteins enabled their affinity capture on anti-FLAG agarose beads (Figure 1B). Full-length NRPD1 and NRPD1ΔCTD copurify with equivalent amounts of NRP(D/E)2, the Pol IV second-largest subunit, and RDR2 (Figure 1B) indicating that Pol IV subunit assembly and RDR2 association do not require the CTD.

Detection of the recombinant NRPD1 proteins in isolated cell nuclei using anti-FLAG antibodies revealed that full-length NRPD1 and NRPD1ΔCTD are both detectable within punctate foci (Figure 1C), consistent with previous studies of native NRPD1 (33). The percentage of nuclei displaying this pattern was somewhat lower for NRPD1ΔCTD than for full-length NRPD1 (53 versus 74%), but the significance of this observation is unclear.

To test whether the CTD is needed for catalytic activity, Pol IV–RDR2 complexes were affinity purified, by virtue of the FLAG epitope tags engineered at the C-termini of NRPD1 or NRPD1ΔCTD, and assayed side-by-side for Pol IV transcription, Pol IV termination and coupling of Pol IV termination with RNA second strand synthesis by RDR2. As reported recently, Pol IV engaged in transcription of a single-stranded DNA template terminates 12–16 after encountering a basepaired non-template DNA strand (8). Moreover, termination induced in this manner is required for RDR2 to use the Pol IV transcript as a template and synthesize a complementary RNA strand, thus generating a dsRNA that can be diced by DCL3 (8). Transcription by Pol IV and RDR2 can be monitored using a 51 nt DNA template lacking thymidines (T-less template) to which a 16 nt RNA primer and 28 nt non-template DNA strand have been annealed (8), as depicted in the diagram of Figure 1D (see also Supplementary Figure S1B for nucleic acid sequences). By end-labeling the RNA primer with ^32^P, first-strand transcripts generated by Pol IV can be visualized by autoradiography, following denaturing polyacrylamide gel electrophoresis (Figure 1D, lanes 2–3; see also Supplementary Figure S1A). Alternatively, by using an unlabeled primer to initiate Pol IV transcription, α-^32^P-ATP incorporation can be used to body-label second-strand transcripts synthesized by RDR2 (Figure 1D, lanes 5–6). Because the DNA template strand lacks T’s, A’s cannot be incorporated into first strand transcripts. Instead, U’s present in the first strands template A incorporation into second strands (8). The RDR2 transcripts migrate more slowly than Pol IV transcripts due to sequence-dependent differences in the complementary strands and due to RDR2′s terminal transferase activity, which adds one (and sometimes 2) extra non-templated nucleotide to the 3′ ends of RDR2 transcripts (3,8). Collectively, the results of Figure 1D (see also Supplementary Figure S1A) show that Pol IV assembled using full-length NRPD1 or NRPD1ΔCTD has indistinguishable Pol IV elongation, Pol IV termination and RDR2 coupling activities, indicating that the CTD is not required for the catalytic steps that generate the double-stranded RNA precursors of 24 nt siRNAs (8).

### CTD deletion impacts siRNA and DNA methylation levels *in vivo*

**Figure 2.**
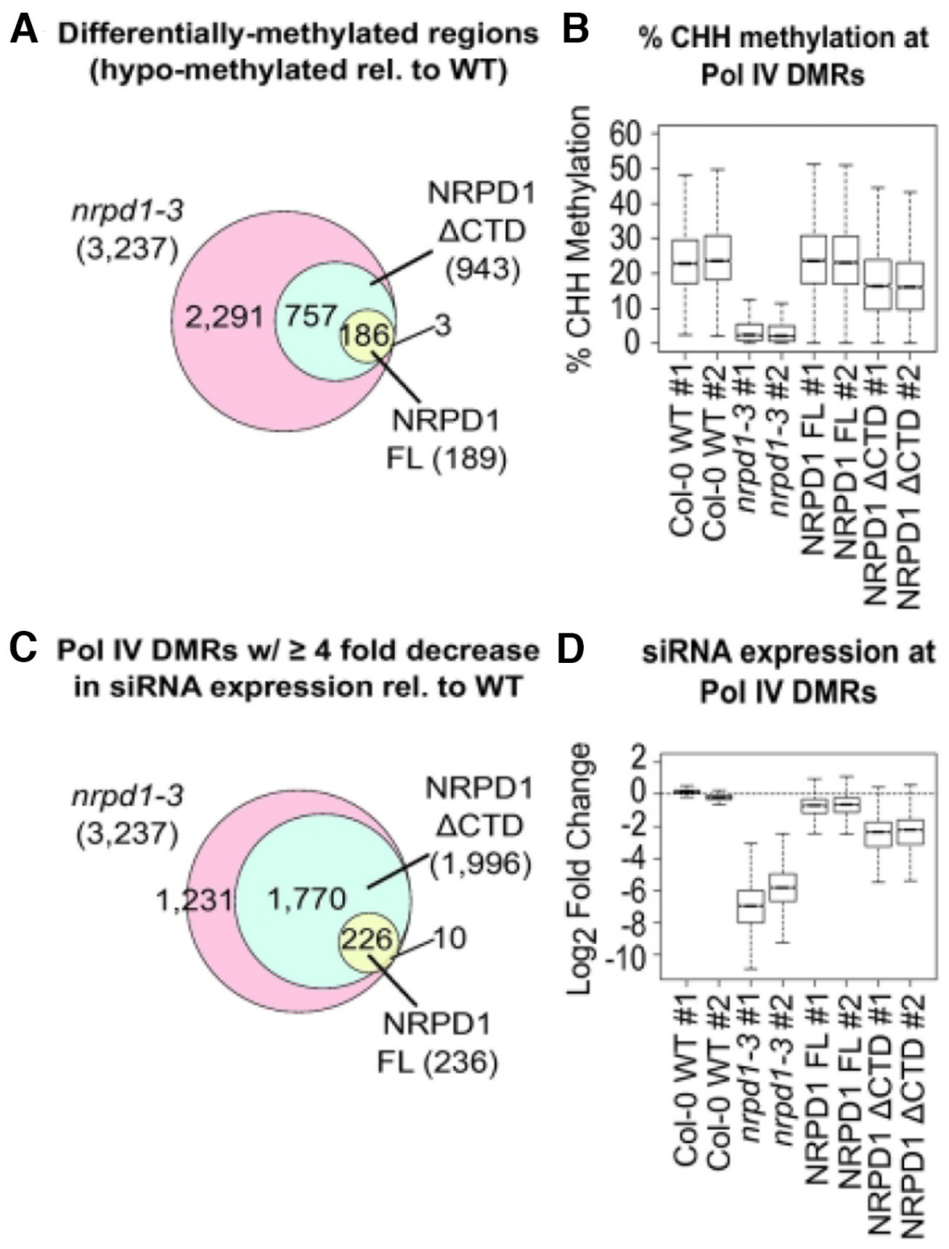
Genome-wide assessment of the abilities of full length NRPD1 and NRPD1ΔCTD to restore DNA methylation and siRNA expression in the *nrpd1-3* null mutant background. (**A**) Venn diagram comparing the number of DMRs (hypo-methylated with respect to WT, Col-0) identified by whole genome bisulfite sequencing of two biologic replicates for each genotype tested (See ‘Materials and Methods’ section). Genomic coordinates of hypo-methylated regions are provided in Supplementary Table S2. (**B**) Box plots summarizing the % methylation for cytosines in the CHH context within the 3237 Pol IV-dependent DMRs. Values for each biologic replicate of each genotype are shown (see Supplementary Table S2 for % methylation values for each DMR for each replicate). (**C**) Venn diagrams comparing the number of Pol IV-dependent DMRs at which siRNA levels 4-fold or more compared to WT levels based on RNA-seq data for two biologic replicates for each genotype. See Supplementary Table S3 for sRNA read counts and calculations. (**D**) Box plots summarizing the log_2_ fold change in siRNA expression relative to WT Col-0 across all 3237 Pol IV-dependent regions for each replicate of each genotype tested. See Supplementary Table S3 for sRNA read counts and calculations.

To assess the importance of the CTD on Pol IV functions *in vivo*, we conducted bisulfite sequencing to map positions of cytosine methylation genome-wide in parallel with small RNA deep sequencing, comparing WT *A. thaliana* (accession Col-0), *nrpd1-3, nrpd1-3* expressing full-length NRPD1 or *nrpd1-3* expressing NRPD1ΔCTD (see Supplementary Table S1 for sequencing statistics). Two independent biological replicates were tested for each genotype. Analysis of the bisulfite sequencing data focused on cytosine methylation in the CHH context, which is a hallmark of RdDM. We first identified differentially methylated regions (DMRs) of WT versus *nrpd1-3* mutants. We then filtered loci for their correspondence to siRNA generating loci, identifying DMRs where 24 nt siRNA levels are reduced at least 4-fold (log_2_ = −2) in *nrpd1-3* mutants relative to WT. Using these parameters, 3237 loci are sites of siRNA synthesis that become significantly hypo-methylated in *nrpd1-3* mutants relative to WT (Figure 2A and C; Supplementary Tables S2 and 3).

Expression of full length NRPD1 in the *nrpd1-3* mutant background restored methylation above cutoff levels at >94% of the 3237 DMRs, but with 189 remaining hypo-methylated (Figure 2A). Because Pol IV recruitment signals are epigenetically inherited, and involve cytosine methylation (30), the 6% of DMRs that remain hypomethylated upon rescue with full length NRPD1 might include loci whose loss of methylation in the *nrpd1-3* mutant background impaired subsequent Pol IV recruitment. Expression of NRPD1ΔCTD restored methylation at 71% of the DMRs, but 943 DMRs remained hypo-methylated (Figure 2A). Collectively, these results show that CTD-dependence of DMR methylation is locus-specific.

CHH-context cytosines present in the 3,237 DMRs show ∼22–24% methylation levels in WT Col-0 plants but only 2–3% methylation in the *nrpd1-3* mutant (Figure 2B). Expression of full-length NRPD1 in the *nrpd1-3* mutant background restores overall % CHH methylation to levels indistinguishable from WT in both replicates (Figure 2B). In plants expressing NRPD1ΔCTD in the *nrpd1-3* mutant background, CHH methylation is restored to 16–17%, indicating substantial rescue of methylation but not full rescue (Figure 2B). Collectively, the results of Figures 2A and B indicate that in the absence of the CTD, Pol IV function in RdDM is compromised but not abolished.

Pol IV’s role in RdDM is to generate precursors of 24 nt siRNAs, thus small RNA-seq data were analyzed to examine CTD effects on siRNA levels. Examining siRNA read counts at the 3237 DMRs revealed that expression of full length NRPD1 in the *nrpd1-3* mutant background restored siRNA levels above the cutoff level (log_2_ = −2) at ∼93% of the loci, but with 236 loci having siRNA levels still below the cutoff (Figure 2C and Supplementary Table S3). In contrast, expression of NRPD1ΔCTD restored siRNA levels at only ∼38% of loci (1241/3237), with 1996 loci having siRNA levels at least 4-fold lower than WT (Figure 2C and Supplementary Table S3). Similar proportional effects on siRNA levels were found when examining 24 nt siRNA clusters genome-wide, irrespective of coverage with bisulfite sequencing (Supplementary Figure S2). This suggests that our decision to focus on regions for which there is both strong bisulfite sequencing and strong sRNA sequencing coverage does not yield results that differ from general trends.

Quantitative assessment of small RNA-seq read numbers shows that in *nrpd1-3* mutants, siRNA levels fall to ∼1% of WT levels (WT/mutant log_2_ median values of −6.9 and −5.8 for the two *nrpd1-3* replicates) (Figure 2D and Supplementary Table S3). Expressing full length NRPD1 in the *nrpd1-3* mutant background restores siRNA levels to ∼62% of WT (transgenic line log_2_ = −0.7 for both replicates) (Figure 2D and Supplementary Table S3). In contrast, expressing NRPD1ΔCTD in the *nrpd1-3* background restored siRNA levels to only ∼20% of WT (log_2_ = −2.3 and −2.2 for the replicates) (Figure 2D and Supplementary Table S3). Collectively, the results of Figures 2C and D show that the CTD is not essential for siRNA biosynthesis but has quantitative effects on siRNA levels.

### Relationships between siRNA and CHH methylation levels are locus-specific.

**Figure 3.**
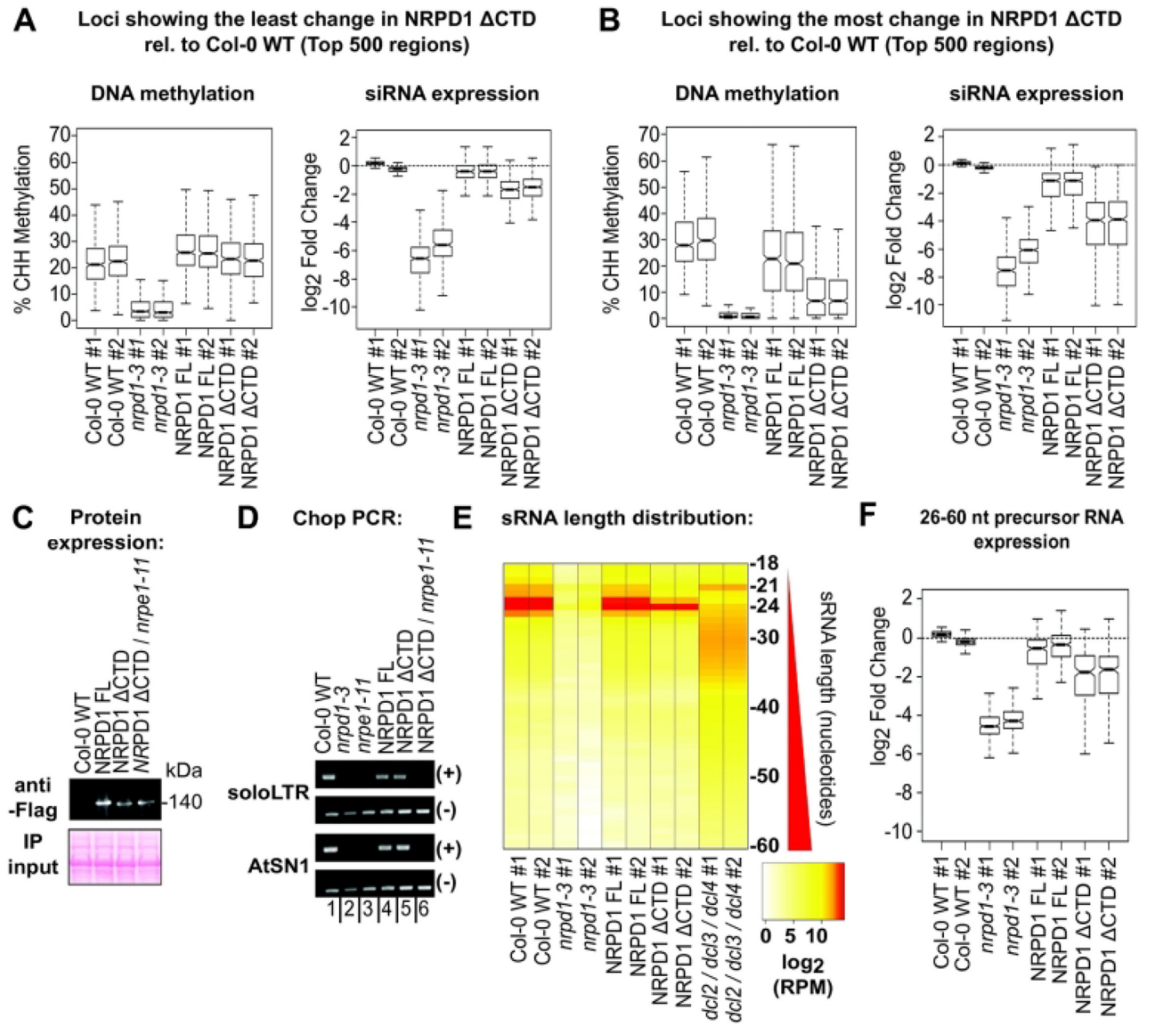
Evidence that Pol IV-dependent siRNAs typically exceed levels needed for full RdDM. Loci that undergo Pol IV-dependent DNA methylation were ranked based on the degree to which NRPD1 ΔCTD and WT Col-0 differ. The top 500 loci at which methylation levels are restored to the highest extent were analyzed in (**A**) and the 500 loci at which methylation levels were least restored are shown in (**B**). In A and B, the left panel shows box plots summarizing % methylation and the right panel shows summaries of log_2_ fold changes in siRNA levels at the 500 loci for the two replicates for each genotype. See Supplementary Tables S4 and 5 for sRNA fold change values for individual loci included in the analyses. (**C**). Immunoblot affinity captured NRPD1 proteins in different genetic backgrounds. Equal input levels are demonstrated by Ponseau S staining of proteins blotted to nitrocellulose membranes. (**D**) Chop-PCR analysis of cytosine methylation at AluI or HaeIII restriction endonuclease cut sites that undergo RdDM in WT (Col-0) plants. (+) indicates that PCR was conducted on genomic DNA that was digested by both enzymes and (−) indicates PCR conducted on uncut DNA. Failure to detect a PCR product in the (+) digest fraction is indicative of loss of methylation. (**E**) Heatmap depicting the distribution of sRNA size classes within 3237 loci dependent on Pol IV for methylation and siRNA expression. Values depicted are the log_2_ of reads per million mapped for each size class. See Supplementary Table S6 for RPM values of each size class. (**F**) Box plot showing the change in levels (log_2_ scale) of RNAs sized 26–60 nt in regions where Pol IV-dependent siRNA expression and RdDM occurs. Regions that had a minimum coverage of 10 reads for 26–60 nt RNAs in each Col-0 replicate were included in the analysis. See Supplementary Table S7 for regions and fold change values.

Our finding that deletion of the Pol IV CTD has a large impact on siRNA levels but a more modest impact on DNA methylation levels genome-wide prompted us to examine the relationship between siRNA levels and DNA methylation at specific loci. We identified 500 loci at which methylation is severely depleted in *nrpd1-3* mutants yet is completely rescued by either full-length NRPD1 or NRPD1ΔCTD (Figure 3A). Interestingly, siRNA levels at these 500 loci are not fully restored by NRPD1ΔCTD despite the full restoration of methylation; instead, siRNA levels average ∼35% of WT levels (−1.5 log_2_ change) (Figure 3A and Supplementary Table S4). Thus, at these loci, at least two-thirds of the siRNAs normally produced in WT cells appear to be dispensable for what appears to be full methylation based on quantitative measures.

We next examined the 500 loci where methylation lost in the *nrpd1-3* mutant is least rescued by expressing NRPD1ΔCTD. At these loci, siRNAs levels in the NRPD1ΔCTD plants are only ∼6% of WT (−4 log_2_ fold change) (Figure 3B and Supplementary Table S5). Thus, very low siRNA levels are predictive of low cytosine methylation levels, in keeping with our current understanding of the RdDM process. The data of Figure 3A and B suggest that siRNA levels that are ∼35% of WT may be sufficient whereas siRNA levels that are only ∼6% of WT are apparently below the threshold level needed for full RdDM.

We considered the possibility that the NRPD1ΔCTD subunit may enable Pol IV function via an alternative RdDM pathway. As a test of this hypothesis, we crossed *nrpd1* NRPD1ΔCTD plants with a *nrpe1-11* mutant defective for Pol V and identified *nrpe1 nrpd1* NRPD1ΔCTD plants among the F2 progeny. The expression level of the NRPD1ΔCTD protein was unaffected by the *nrpe1* mutation (Figure 3C). We next assessed DNA methylation at well-studied transposable element loci, *AtSN1* and *soloLTR* using methylation-sensitive restriction endonuclease digestion followed by PCR (Chop-PCR; Figure 3D). DNA methylation is lost at the test sites of both loci in *nrpd1-3* and *nrpe1-11* mutants (compare lanes 1–3) but is restored in *nrpd1* mutants that express full length or ΔCTD versions of NRPD1 (lanes 4 and 5). However, methylation is not restored if *nrpe1* is also mutant (lane 6), indicating that NRPD1ΔCTD-dependent restoration of methylation is still Pol V-dependent, consistent with the known RdDM pathway.

### The CTD is not required for siRNA precursor processing

Reduced siRNA levels in *nrpd1* plants expressing NRPDΔCTD might potentially reflect impaired processing of Pol IV/RDR2-dependent precursors (P4R2 RNAs) into siRNAs. P4R2 RNAs are mostly ∼30–50 nt in length (3,6) and are present at low levels in WT plants due to their efficient processing by Dicer endonuclease activity (3). However, if siRNA processing is impaired, as in Dicer mutants, P4R2 RNAs accumulate (3,6,42). To test whether P4R2 RNAs accumulate in plants expressing NRPD1ΔCTD, we compared the size distribution of sRNAs in the DMRs of WT, *nrpd1-3, nrpd1-3* plants expressing either full-length NRPD1 or NRPD1ΔCTD, and Dicer *dcl2/dcl3/dcl4* triple mutants (3). In WT plants, the siRNA size distribution, displayed as a heat map, shows a strong signal at 24 nt and very low signals for RNAs longer than 25 nt (Figure 3E and Supplementary Table S6). These 24 nt siRNAs are virtually eliminated in *nrpd1-3* mutants but are restored upon expression of full-length NRPD1 or NRPD1ΔCTD (Figure 3E and Supplementary Table S6). No evidence for longer un-diced precursors was detected, as observed for *dcl2/dcl3/dcl4* triple mutants in which P4R2 RNAs of ∼24–37 nt accumulate (Figure 3E and Supplementary Table S6) (3). Collectively, these results indicate that siRNA processing is not dependent on the CTD of NRPD1, suggesting that the low levels of siRNAs in NRPD1ΔCTD plants reflects reduced precursor RNA synthesis. Consistent with this hypothesis, examination of putative P4R2 precursor RNAs in the 26–60 nt size range, which are detectable at low levels at some loci in WT plants (3), revealed that precursor levels are reduced to the same extent as siRNA levels at these loci in NRPD1ΔCTD plants (Figure 3F and Supplementary Table S7).

### CTD effects on transcriptional silencing are locus-specific

**Figure 4.**
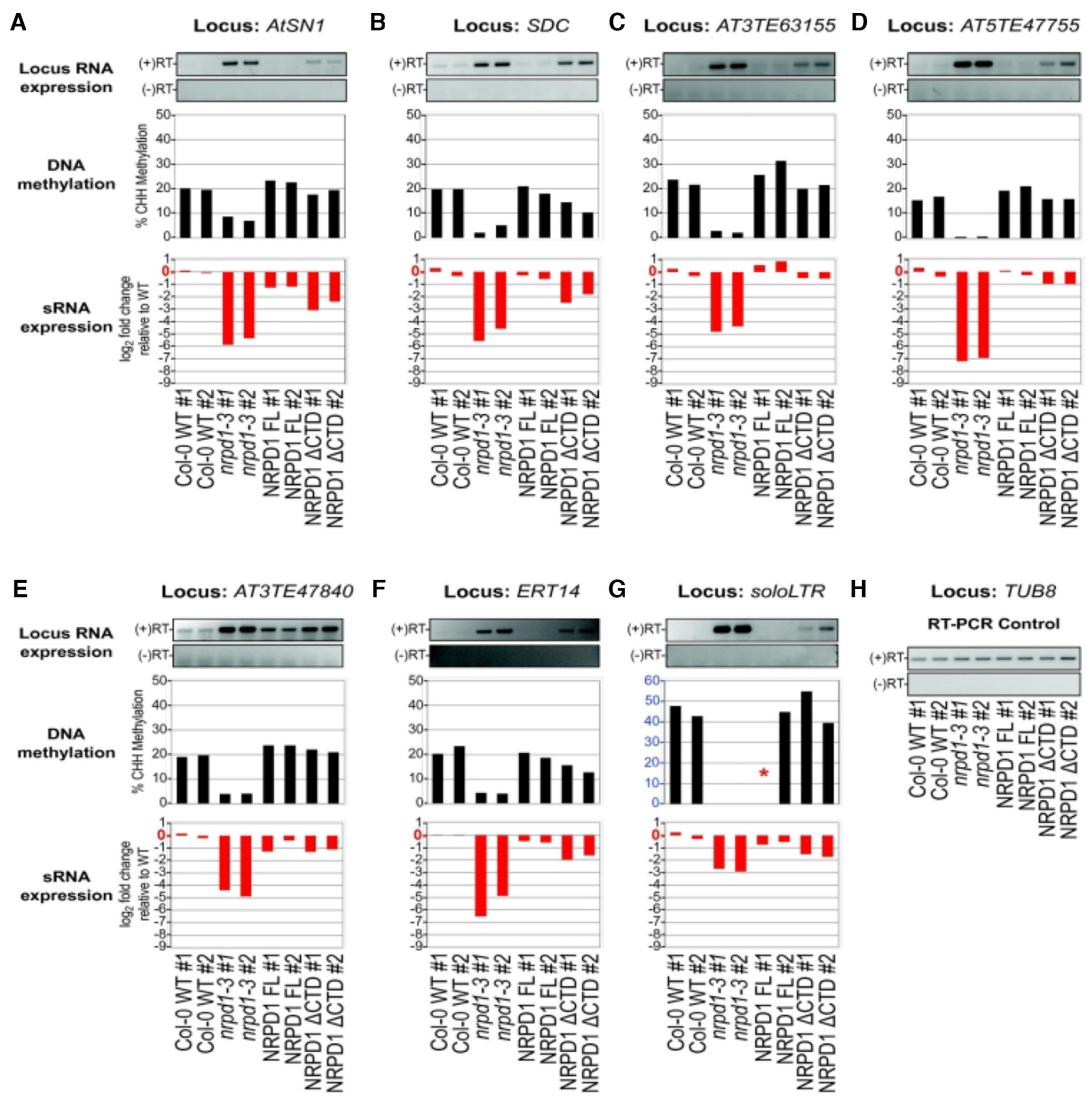
DNA methylation and sRNA expression profiles of genes where Pol IV-dependent transcriptional silencing is not restored by expressing NRPD1ΔCTD. RNA expression at loci that require Pol IV for transcriptional silencing were analyzed by RT-PCR, in parallel with analysis of their CHH methylation and siRNA levels. (**A**) *AtSN1* (*AT3TE63860*); (**B**) *SDC* (*AT2G17690*); (**C**) *AT3TE63155*; (**D**) *AT5TE47755*; (**E**) *AT3TE47840*; (**F**) *ERT14 (AT2G01422)*; (**G**) *soloLTR (AT5TE35950)*; (**H**) *TUB8*. For each locus, the top panel shows RT-PCR products visualized on agarose gels, the middle panel shows % CHH methylation and the bottom panel shows the log_2_ fold change in siRNA expression relative to the average expression of both Col-0 WT replicates. *TUB8* (tubulin) is a Pol II transcribed gene that is not subject to RdDM and serves as an RNA input control for RT-PCR assays. There was not adequate bisulfite sequencing coverage in the NRPD1 FL replicate #1 to assess % methylation for this line at the *soloLTR* locus (indicated by *). Note also that the scale for the % methylation histogram of *soloLTR* is different than for other loci to account for higher levels of methylation at this site.

**Figure 5.**
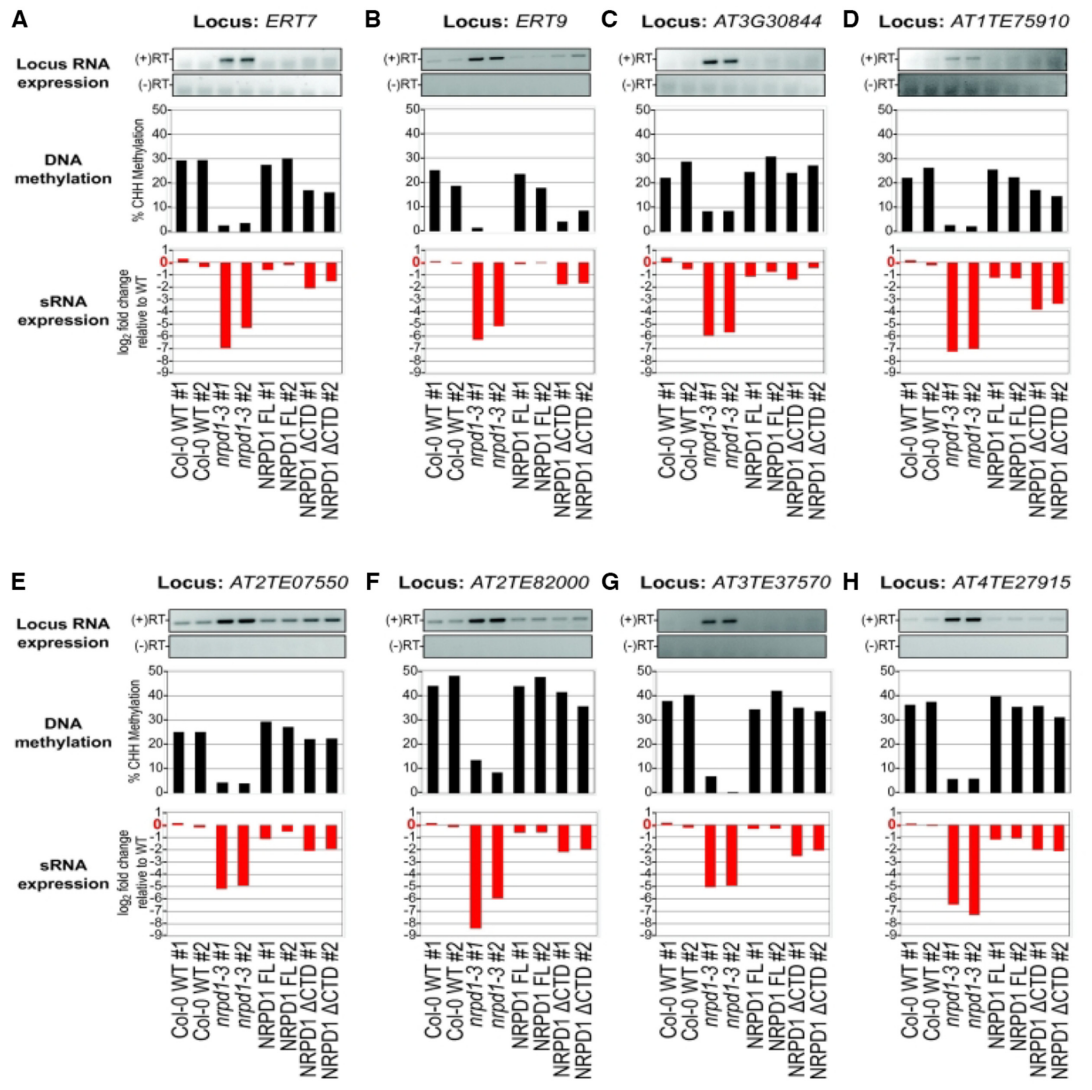
DNA methylation and sRNA expression profiles of genes where Pol IV-dependent transcriptional silencing is restored by expressing NRPD1ΔCTD. (**A**) *ERT7* (*AT3G28899*); (**B**) *ERT9* (*AT5G24240*); (**C**) *AT3G30844*; (**D**) *AT1TE75910*; (**E**) *AT2TE07550*; (**F**) *AT2TE82000*; (**G**) *AT3TE37570*; (**H**) *AT4TE27915*. For each locus, the top panel shows RT-PCR products visualized on agarose gels, the middle panel shows % methylation, and the bottom panel shows the log_2_ fold change in siRNA expression relative to the average expression of both Col-0 WT replicates. The input control for the RT-PCR reactions is shown in Figure 4H.

To investigate the importance of the NRPD1 CTD in transcriptional silencing, we examined previously characterized loci whose silencing is known to be dependent on Pol IV (30). Data for 15 such loci, showing their expression status, CHH methylation levels and siRNA levels are shown in Figures 4 and 5. For all 15 loci, transcripts are not detected (or detected at low levels) in WT plants but are derepressed in *nrpd1-3* mutants and are re-silenced upon expression of full-length NRPD1. Loci grouped in Figure 4 are those for which expression of NRPD1ΔCTD partially, but incompletely, restores silencing. By contrast, the loci grouped in Figure 5 are those for which expression of NRPD1ΔCTD fully restores silencing. For both sets of loci (Figures 4 and 5), a generalization is that expression of NRPD1ΔCTD does not fully restore siRNA nor cytosine methylation to WT levels. However, transcriptional silencing was restored, despite inefficient restoration of methylation, at some loci, such as *ERT7* and *ERT9* (Figure 5A and B). In other cases, cytosine methylation levels were restored to near WT levels by NRPD1ΔCTD expression, yet silencing was not completely restored (Figure 4A, C, D and G).

We considered the possibility that single-nucleotide, position-specific changes in small RNA or DNA methylation frequency might go unnoticed in sliding-window analyses that only count RNAs or methylcytosines within a given interval. However, displaying sRNAs and methylcytosine data aligned to DNA sequences using JBrowse (43) revealed no evidence that this is the case (see Supplementary Figure S3 for JBrowse images corresponding to the loci examined in Figure 4 and see Supplementary Figure S4 for loci examined in Figure 5).

### CTD-dependent loci overlap with loci dependent on CLSY1/2 and SHH1

**Figure 6.**
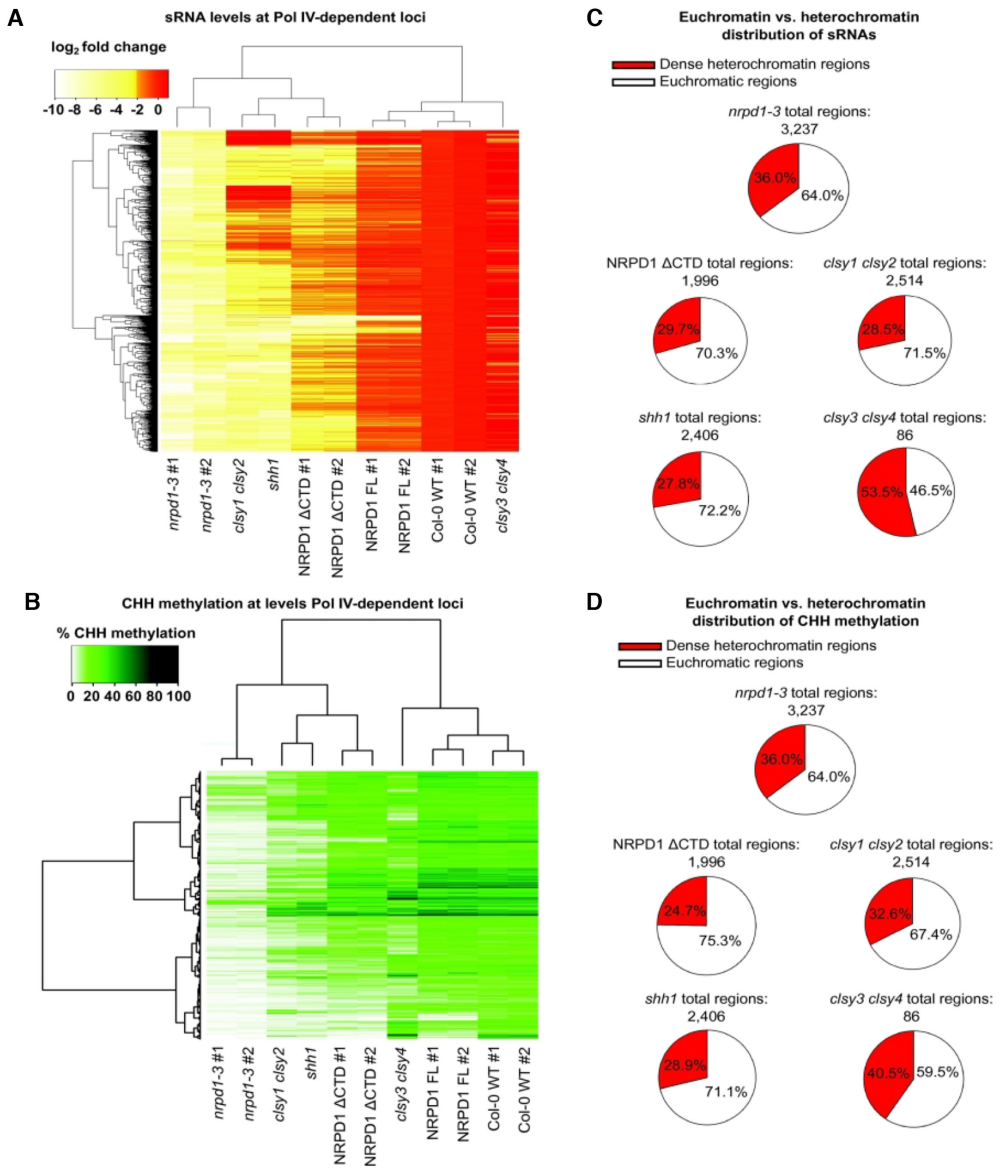
Comparison of Pol IV CTD-dependent loci with RdDM loci dependent on CLSY1-4 and SHH1/DTF1. (**A**) Hierarchical clustering and heatmap display showing log_2_ fold changes in siRNA levels in various mutant genotypes relative to WT Col-0 at the 3237 Pol IV-dependent regions defined in this study. See Supplementary Table S3 for sRNA read counts and calculations. (**B**) Heatmap and hierarchical clustering analysis for % CHH methylation across all 3237 Pol IV-dependent regions. See Supplementary Table S2 for % CHH methylation values. (**C**) Pie charts denoting the distribution between dense heterochromatic regions (red) and more euchromatic chromosome arms (white) for loci found to have a minimum log_2_ change of at least −2 relative to WT in the indicated genotypes. (**D**) Pie charts denoting the distribution of loci found to have a significant decrease in %CHH methylation levels relative to Col-0 WT in the indicated genotype between dense heterochromatic regions (red) and chromosome arms (white). See Methods for chromosomal coordinates defining dense heterochromatin.

Pol IV recruitment to target loci is thought to be mediated by SAWADEE HOMEODOMAIN HOMOLOG 1 (SHH1) (26) and CLASSY family proteins 1 through 4, with CLSY1 and CLSY2 acting at loci distinct from those requiring CLSY3 and CLSY4 (27). To determine if the absence of the Pol IV CTD might phenocopy loss of CLSY or SHH1 activity, we performed hierarchical clustering analyses, comparing the changes, relative to WT, in siRNA levels at Pol IV-dependent loci (Figure 6A). Plants expressing Pol IV lacking the CTD cluster to some extent with *shh1* and *clsy1clsy2* double mutants, but not with *clsy3 clsy4* double mutants (Figure 6A and Supplementary Table S3). However, the heat map profile for *nrpd1ΔCTD* is distinct from the profiles of *shh1* or *clsy1clsy2*. Similarly, clustering analyses for CHH methylation (Figure 6B and Supplementary Table S2) shows that *clsy1clsy2* and *shh1* mutants have stronger loss of methylation phenotypes than plants lacking the Pol IV CTD at most affected loci.

CLSY1/2 and SHH1-dependent Pol IV target regions have been shown to be enriched within the mostly euchromatic arms of *A. thaliana* chromosomes whereas CLSY3/4-dependent regions are enriched in pericentromeric regions of dense heterochromatin (27). Pol IV target loci dependent on the CTD show a euchromatin/heterochromatin distribution that is similar to that of CLSY1/2 and SHH1-dependent loci and unlike the distribution observed for CLSY3/4-dependent loci, when either siRNA or CHH effects are considered (Figures 6C and D). Collectively, the data of Figure 6 indicate that the deletion of the Pol IV CTD does not phenocopy the loss of *CLSY1* and *2* or *SHH1* yet affects Pol IV recruitment or activity in a similar chromatin context.

## DISCUSSION

Unlike the DeCL domain of the Pol V CTD, whose deletion essentially eliminates Pol V transcripts and RdDM, the DeCL domain of the Pol IV CTD is not essential for Pol IV function, *in vitro* or *in vivo*. Pol IV missing the CTD can still generate precursor RNAs that are processed into siRNAs *in vivo*, and RdDM ensues, indicating that Pol IV is being recruited to its target sites. However, siRNA accumulation and cytosine methylation attributable to RdDM are reduced at target sites in the absence of the CTD, indicating that the CTD is important in some way. The enzyme's core catalytic activity, or ability to associate with RDR2 to achieve coupled Pol IV-RDR2 synthesis of dsRNA, does not appear to be affected by the CTD based on *in vitro* transcription assays (see Figure 1 and Supplementary Figure S1). However, the possibility that the CTD plays a role in Pol IV transcription of chromosomal DNA cannot be ruled out. Less efficient recruitment to target loci, slower elongation, or impaired termination and coupling with RDR2 in the context of chromatin are potential steps of the Pol IV transcription cycle that might involve the CTD. Unfortunately, assays for these individual steps of the Pol IV transcription cycle in vivo do not yet exist, such that a mechanistic understanding of the deficiencies apparent when the CTD is deleted is not yet feasible.

The most dramatic consequence of deleting the Pol IV CTD is the 80% reduction in siRNA levels, genome-wide, relative to WT. By contrast, CHH methylation frequency attributable to RdDM is not impacted as severely as siRNA levels yet falls from a frequency of ∼23% CHH methylation in WT to ∼17% in the absence of the CTD (see Figure 2). Many loci show no decrease in methylation relative to WT in plants expressing NRPD1 missing the CTD, despite lower siRNA levels (see Figure 3A). Collectively, the results demonstrate that the relationship between siRNA levels and DNA methylation is not linear, consistent with observations made for specific loci in early studies of RdDM (44). Instead, the data support the hypothesis that there is a threshold level of siRNAs needed for full methylation of a given locus, which could potentially vary depending on the chromatin context. In general, siRNA levels in WT plants appear to exceed these thresholds.

An interesting group of loci identified in our study had CHH methylation restored to WT levels by NRPD1ΔCTD yet transcriptional silencing was not completely restored (e.g. see Figure 4A, C, D, E and G). One simple explanation might be that quantitative measures of CHH methylation restoration do not capture qualitative effects of methylating specific cytosines critical for silencing. However, the possibility that siRNAs may have functions separate from, or in addition to, guiding DNA methylation cannot be ruled out, in analogy to the siRNA guidance of repressive histone modifications in eukaryotes that do not methylate their DNA (45,46).

A difference between Pol IV-regulated loci dependent on CLSY1 or CLSY2 versus CLSY3 and CLSY4 is that CLSY1/2-dependent loci are more prevalent in chromosome arms whereas CLSY3 and CLSY4 are more important in pericentromeric regions composed of dense heterochromatin (27). SHH1-dependent loci are highly correlated with CLSY1/2-dependent loci (27), and this is readily apparent in their highly similar heatmap profiles in Figure 6. Loci dependent on the Pol IV CTD have a chromosome arm versus pericentromeric localization profile similar to SHH1 and CLSY1/2 (Figure 6C and D). However, the heatmap profiles for the CTD deletion replicates are distinct from those of CLSY1/2 and SHH1, suggesting that the CTD affects processes distinct from steps catalyzed by CLSY-SHH1. Developing an understanding of the Pol IV transcription cycle at a biochemical level will likely be key to understanding these steps in the context of different chromatin states.

## DATA AND REAGENT AVAILABILITY

Plant strains are available upon request. Deep sequencing data generated in this study have been deposited in NCBI’s Gene Expression Omnibus (47) and are accessible through GEO Series accession number GSE95825. Whole genome sRNA and bisulfite sequencing statistics and data accessions numbers are provided in Supplementary Table S1. Genomic coordinates and numerical values for % CHH methylation of Pol IV-dependent DMRs is provided in Supplementary Table S2. Numerical values for sRNA read counts and all calculations for sRNA analyses at Pol IV-dependent loci are provided in Supplementary Table S3. Genomic coordinates and % CHH methylation and small RNA values for 500 loci whose methylation is most or least affected by deletion of the CTD are provided in Supplentary Tables S4 and 5. sRNA read counts for each nt size class (18–60 nt) across all Pol IV dependent loci are in Supplementary Table S6. Read count values and calculations for siRNA precursor RNAs are in Supplementary Table S7. Oligonucleotides used in the study are provided in Supplementary Table S8.

## Supplementary Material

gkz615_Supplemental_Files

## Notes

Present address: Jeremy R. Haag, Bayer Crop Science, 700 West Chesterfield Parkway, Chesterfield, MO 63017, USA.

Present address: Jered M. Wendte, Department of Genetics, University of Georgia, 120 East Green Street, Athens, GA 30602, USA.
