# Supplementary material for: The Pol IV largest subunit CTD quantitatively affects siRNA levels guiding RNA-directed DNA methylation
Source: Nucleic Acids Res. 2019 Jul 22;47(17):9024–36. doi: 10.1093/nar/gkz615 (PMC6753486; doi:10.1093/nar/gkz615)
Supplement: gkz615_Supplemental_Files [file gkz615_supplemental_files.zip › Supplement minus tables.pdf]

Supplemental Information

- includes Methods, four Figures and eight Tables.

Supplemental methods (related to Figure S1)

In vitro transcription was conducted using Pol IV or Pol II affinity captured on anti-FLAG agarose resin (Sigma) by virtue of FLAG epitope tags engineered into NRPD1 or NRPB1 (4,25). Polymerase-bound resin was washed once with CB100 buffer (100 mM potassium acetate, 25 mM HEPES-KOH pH 7.9, 20% glycerol, 0.1 mM EDTA, 0.5 mM DTT, 1 mM PMSF), then resuspended in a mix of 50 µL CB100 buffer and 50 µL 2x transcription reaction buffer (120 mM ammonium sulfate, 40 mM HEPES-KOH pH 7.6, 20 mM magnesium sulfate, 20 µM zinc sulfate, 20% glycerol, 0.16 U/µL RNaseOUT, 20 mM DTT , 2 mM ATP, 2 mM UTP, 2 mM GTP, 0.08 mM CTP, 0.2 mCi/mL alpha ^{32}P-CTP and 4 pmols of template). Transcription reactions were conducted at room temperature for 60 minutes on a rotating mixer and stopped by addition of 50 mM EDTA and heating at 75˚C for five minutes. Transcription products were enriched using PERFORMA spin columns (EdgeBio) and precipitated using 1/10 volume of 3M sodium acetate, pH 5.2, 20 µg glycogen and 2 volumes isopropanol at -20˚C overnight. Radioactive RNA transcripts were resolved on 15% denaturing polyacrylamide gels, transferred to Whatman 3MM filter paper, dried under vacuum and visualized by phosphorimaging.

Figure S1

A. Primer initiated Pol IV (+/- CTD) vs. Pol II transcription, with 11th subunit immunoblot showing relative polymerase quantities

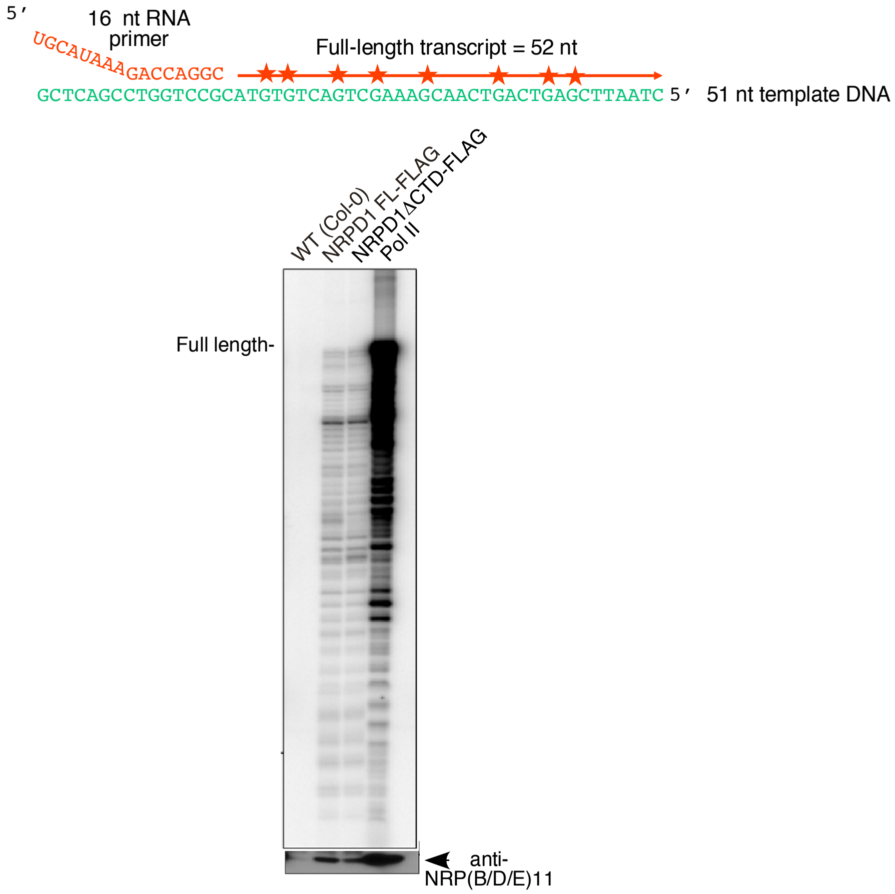

B. Sequences of T-less template, nontemplate strand and RNA primer used in the transcription experiments of Figure 1D

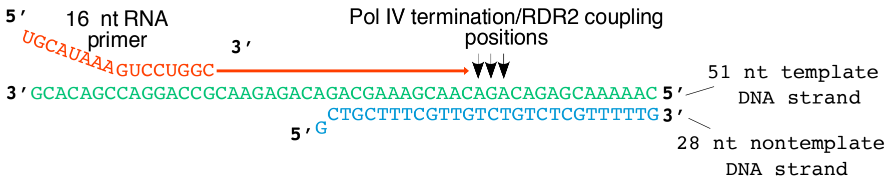

Figure S1. In vitro transcription by Pol IV and Pol IVΔCTD in comparison to Pol II.
A. Primer initiated Pol IV (+/- CTD) vs. Pol II transcription, with 11th subunit immunoblot showing relative polymerase quantities. The transcriptional activities of Pol IV assembled using full length NRPD1-FLAG or NRPD1ΔCTD-FLAG were compared to one another and to Pol II, using a 51 nt DNA template annealed to a 16 nucleotide RNA primer that forms 8 bp with the template, as depicted in the diagram. Transcripts were body-labeled using alpha-32P-labeled CTP and visualized by autoradiography following denaturing polyacrylamide gel electrophoresis. The bottom panel shows an immunoblot in which equal aliquots of affinity-purified proteins were subjected to SDS-PAGE, electroblotted to a PVDF membrane and probed with an antibody raised against NRP(B/D/E)11, a subunit common to Pol II, Pol IV and Pol V.
B. Sequences of the T-less template, nontemplate DNA strand and RNA primer used for the transcription experiments of Figure 1D.

Wendte et. al Figure S2

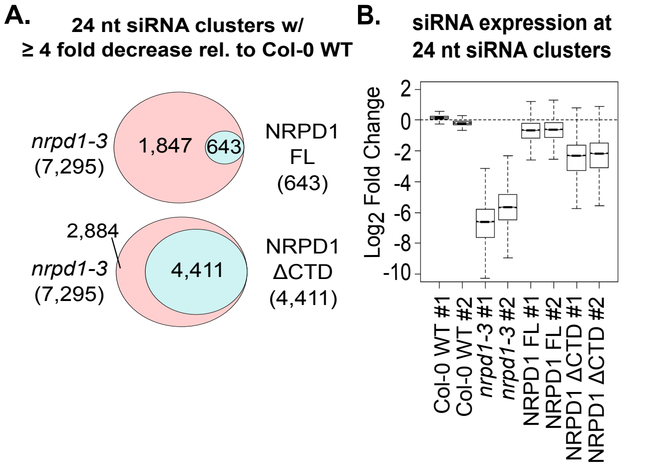

Figure S2. Genome-wide assessment of the abilities of full length NRPD1 and NRPD1ΔCTD to restore siRNA expression at 24nt siRNA clusters in the nrpd1-3 null mutant background.

A. Venn diagrams comparing the number of Pol IV-dependent 24 nt siRNA clusters at which siRNA levels 4-fold or more compared to wild-type levels based on RNA-seq data for two biologic replicates for each genotype.

B. Box plots summarizing the log2 fold change in siRNA expression relative to wild-type Col-0 across all 7,295 Pol IV-dependent 24 nt siRNA clusters identified genome-wide for each replicate of each genotype tested.

Wendte et. al Figure S3

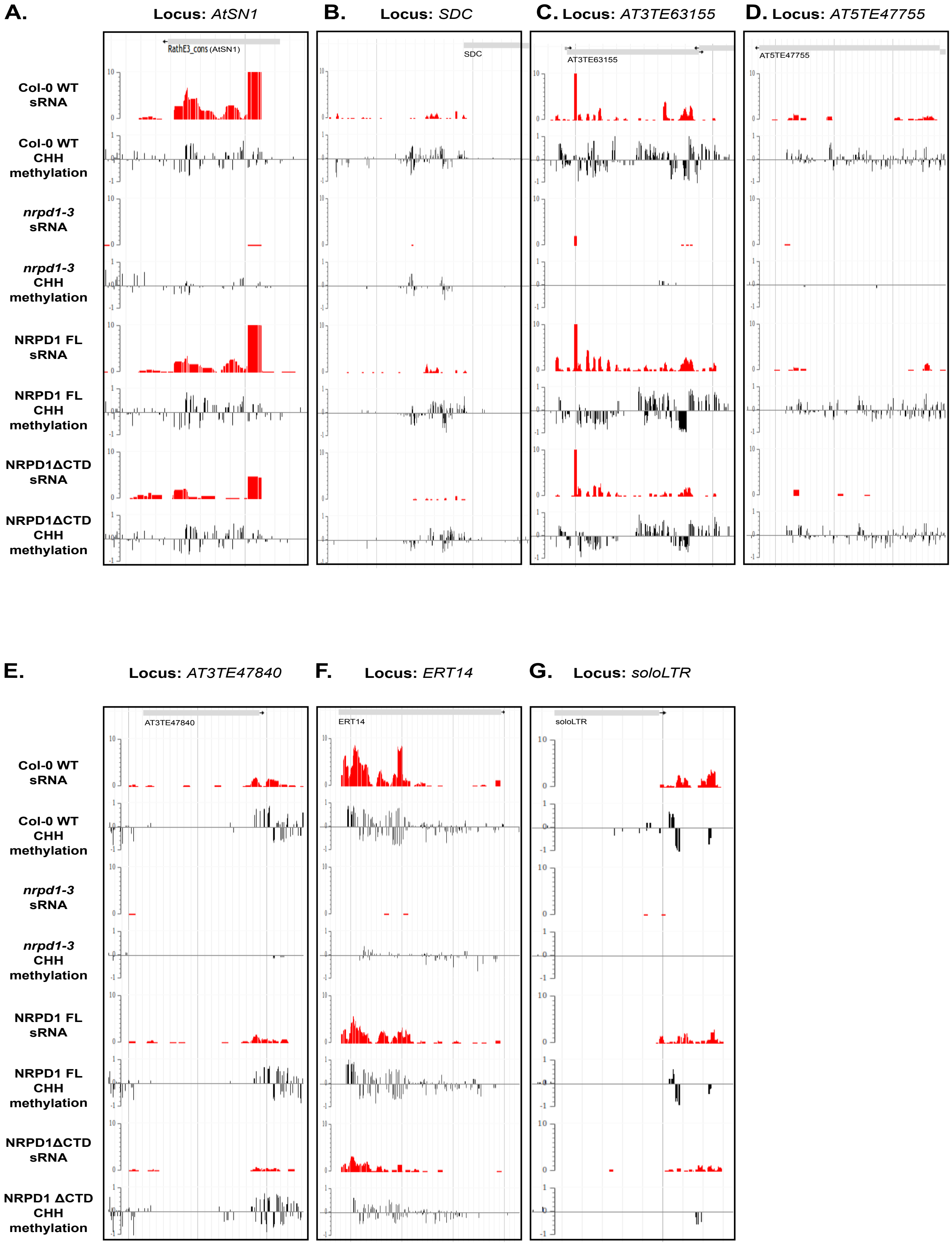

Figure S3. JBrowse screenshots of sRNA and bisulfite sequencing data for loci that remain derepressed in plants expressing NRPD1ΔCTD. Related to Figure 4. A. AtSN1 (AT3TE63860); B. SDC (AT2G17690); C. AT3TE63155; D. AT5TE47755; E. AT3TE47840; F. ERT14 (AT2G01422); G. soloLTR (AT5TE35950). Shown for each locus are tracks corresponding to 24 nt siRNA expression (in reads per million mapped) and percent CHH methylation identified by genome-wide sRNA and bisulfite sequencing of Col-0, nrpd1-3 or nrpd1-3 plants expressing either full length NRPD1 (NRPD1 FL) or NRPD1ΔCTD.

Wendte et. al Figure S4

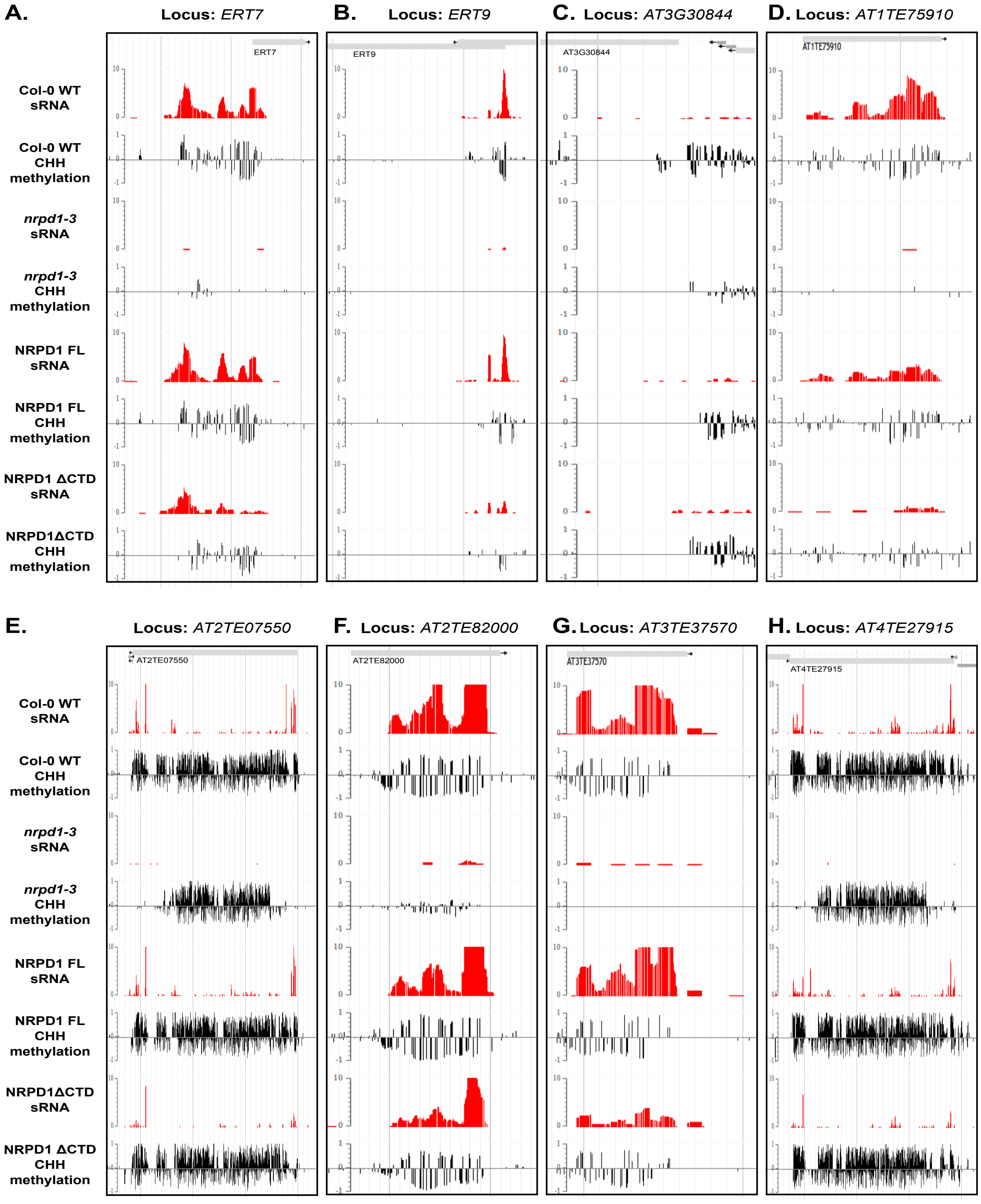

Figure S4. JBrowse screenshots of sRNA and bisulfite sequencing data for loci where transcriptional repression is restored in plants expressing NRPD1ΔCTD. Related to Figure 5. Loci analyzed include A. ERT7 (AT3G28899); B. ERT9 (AT5G24240); C. AT3G30844; D. AT1TE75910; E. AT2TE07550; F. AT2TE82000; G. AT3TE37570; H. AT4TE27915. Shown for each locus are tracks corresponding to 24 nt siRNA expression (in reads per million mapped) and percent CHH methylation identified by genome-wide sRNA and bisulfite sequencing of Col-0, nrpd1-3, or nrpd1-3 plants expressing either full length NRPD1 (NRPD1 FL) or NRPD1ΔCTD.

Table S1. sRNA and bisulfite sequencing statistics

Table S2. % CHH methylation values at Pol IV DMRs. Related to Figure 2 and 6.

Table S3. sRNA read counts and calculations for Pol IV DMRs. Related to Figure 2 and 6.

Table S4. sRNA sequencing log2 fold change values and % CHH methylation values for the top 500 regions characterized by the smallest change in % methylation in NRPD1ΔCTD relative to Col-0. Related to Figure 3.

Table S5. sRNA sequencing log2 fold change values and % CHH methylation values for the top 500 regions characterized by the largest change in % methylation in NRPD1ΔCTD relative to Col-0. Related to Figure 3.

Table S6. sRNA size class distributions across Pol IV DMRs. Related to Figure 3.

Table S7. sRNA sequencing log_2 fold change values for Pol IV-dependent precursor RNA (26-60 nt) levels. Related to Figure 3.

Table S8. Oligonucleotides used in this study.
